# Impact of perceived interpersonal similarity on attention to the eyes of same-race and other-race faces

**DOI:** 10.1186/s41235-021-00336-8

**Published:** 2021-11-02

**Authors:** Kerry Kawakami, Justin P. Friesen, Amanda Williams, Larissa Vingilis-Jaremko, David M. Sidhu, Rosa Rodriguez-Bailón, Elena Cañadas, Kurt Hugenberg

**Affiliations:** 1 York University, Toronto, Canada; 2 University of Winnipeg, Winnipeg, Canada; 3 University of Bristol, Bristol, UK; 4 University College London, London, UK; 5 University of Granada, Granada, Spain; 6 Akili Interactive Laboratories, Boston, USA; 7 Indiana University, Bloomington, USA; 8 Canadian Association for Girls in Science, Mississauga, Canada

**Keywords:** Visual attention, Intergroup bias, Social categorization, Similarity, Face perception

## Abstract

One reason for the persistence of racial discrimination may be anticipated dissimilarity with racial outgroup members that prevent meaningful interactions. In the present research, we investigated whether perceived similarity would impact the processing of same-race and other-race faces. Specifically, in two experiments, we varied the extent to which White participants were ostensibly similar to targets via bogus feedback on a personality test. With an eye tracker, we measured the effect of this manipulation on attention to the eyes, a critical region for person perception and face memory. In Experiment 1, we monitored the impact of perceived interpersonal similarity on White participants’ attention to the eyes of same-race White targets. In Experiment 2, we replicated this procedure, but White participants were presented with either same-race White targets or other-race Black targets in a between-subjects design. The pattern of results in both experiments indicated a positive linear effect of similarity—greater perceived similarity between participants and targets predicted more attention to the eyes of White and Black faces. The implications of these findings related to top-down effects of perceived similarity for our understanding of basic processes in face perception, as well as intergroup relations, are discussed.

**Supplementary Information:**

The online version contains supplementary material available at 10.1186/s41235-021-00336-8.

Person perception often begins with face processing (Hugenberg & Wilson, 2013; Zebrowitz, 2006, 2017). Faces are rich sources of social information that provide important cues about others and are critical to regulating social interactions and forming impressions (Argyle & Cook, 1976; Frischen et al., 2007). Although the traditional approach to person perception assumed that early perceptual cues from others’ faces are spontaneously extracted and determine impressions in a bottom-up manner (Brewer, 1988; Fiske & Neuberg, 1990; Kunda & Thagard, 1996; Macrae & Bodenhausen, 2000), more recently, theorists have argued that impressions of others can be simultaneously influenced by bottom-up cues and top-down effects of the perceiver (Freeman & Ambady, 2011; Freeman & Johnson, 2016; Kawakami et al., 2018; see Kawakami et al., 2017, for a review). In particular, they suggest that both the physical features of the target and the expectancies, motivations, attitudes, and prior knowledge of the perceiver interact to influence person perception, even in its early stages (Hugenberg & Bodenhausen, 2004; Ofan et al., 2011; Ratner & Amodio, 2013; Van Bavel et al., 2011). The present experiments contribute to this literature by investigating the impact of a novel top-down factor, perceived interpersonal similarity, on visual attention to the faces of members of the same race and other races.

A well-known adage is that birds of a feather, flock together. Put simply, people are inclined to like and desire interactions with others who are similar. A large literature has investigated this similarity-attraction effect across a variety of interpersonal similarity domains, including personality traits, attitudes, values, physical characteristics, preferred activities, demographic variables, socioeconomic status, occupation, and fleeting subjective experiences (Bond et al., 1968; Byrne, 1961, 1971; Byrne et al., 1967; Curry & Emerson, 1970; DeBruine, 2002; Griffit, 1966; Lemay & Clark, 2008; Montoya & Horton, 2012; Montaya et al., 2008; Murray et al., 2002; Pinel & Long, 2012; Rokeach et al., 1960; Walton et al., 2012).

The goal of the present research was to extend these findings by investigating the impact of similarity on initial visual attention to features on same-race and other-race faces. Such early stages of face perception have been linked to important downstream consequences, including intergroup differences in trust inferences (Lloyd et al., 2017) and emotion identification (Friesen et al., 2019), as well as dehumanization (Cassidy et al., 2017). In the current studies, we focused on how perceived interpersonal similarity influences spontaneous attention to the eyes and whether this process differs with target race. To this end, we first briefly describe similarity-attraction research. Next, we explore the potential impact of similarity on attentional processes in face perception, with a focus on eye gaze. We also discuss whether this process occurs for both same-race faces (i.e., White participants and White targets) and other-race faces (i.e., White participants and Black targets) and the importance of attention to the eyes in both contexts. Finally, we present two studies in which we manipulate perceptions of personality similarity of White participants to White targets (Experiments 1 and 2) and Black targets (Experiment 2) and measure their impact on attention to the eyes using an eye tracker.

## Similarity-attraction effects and attention to the eyes

There is overwhelming evidence for the similarity-attraction effect. Two meta-analyses, summarizing hundreds of studies, indicate that interpersonal similarity between the target and participant increases interpersonal attraction across a variety of dimensions (Montaya et al., 2008; Montoya & Horton, 2012). This process may be influenced by several key variables. In particular, perceived similarity has a more robust effect on attraction than actual similarity (Condon & Crano, 1988; Hoyle, 1993; Klohnen & Luo, 2003; Montaya et al., 2008; Tidwell et al., 2013; West et al., 2014b) and similarity effects are moderated by interaction quantity over time and by culture (Duck & Craig, 1978; Heine et al., 2009; Montaya et al., 2008). Nonetheless, the current literature indicates that, at least among North American participants in the early stages of relationships, greater perceived similarity results in greater attraction and an increased desire to interact with targets.

Although explicit ratings of liking and a desire to interact are two methods to gauge attraction, research on face processing indicates that nonverbal behaviors can also signal attraction. In particular, behaviors related to immediacy and enhancing psychological closeness, such as looking at another’s face, and especially attention to the eyes, are signs of liking and a positive attitude toward the target (DePaulo & Friedman, 1998; Dovidio et al., 1997; Exline, 1971; Hecht & Ambady, 1999; Kleinke et al., 1975; Scherer & Schiff, 1973). Furthermore, eye contact increases as a function of attraction (Mehrabian, 1968) and triggers the experience of social connection (Wirth et al., 2010). In general, people attend more to the eyes when they share feelings of warmth and liking (Kleinke, 1986).

This increase in attention to others’ eyes under conditions of interpersonal liking and immediacy is functional. The eyes play a central role in person judgment and one that is distinct from other facial features (Itier et al., 2006, 2007; Kawakami et al., in press). Because the eyes provide critical data about preferences and approach-avoidance behaviors, finding strategies to increase eye gaze can facilitate interpersonal relations (Adams & Kleck, 2003; Baron-Cohen et al., 1997; Henderson et al., 2005; Macrae et al., 2002). Looking into the eyes of others allows us to better understand their identity (McKelvie, 1976), intentions (Adams & Kleck, 2005; Mason et al., 2004, 2005), capacities (Khalid et al., 2016; Looser & Wheatley, 2010), and emotions (Adams et al., 2009; Baron-Cohen et al., 2001; Friesen et al., 2019; Itier & Batty, 2009; Niedenthal et al., 2010). It is therefore not surprising that perceivers use this information to regulate social interactions (Hessels et al., 2019).

In the present research, we expected that because similarity breeds attraction and a desire to build and sustain a relationship (Byrne, 1961, 1971; Neff & Karney, 2005; West et al., 2014b), and because attention to the eyes increases as function of liking (Exline & Winters, 1965; Kleinke, 1986; Mehrabian, 1968), participants would show greater attention to the eyes of targets perceived to be more similar.

## Impact of similarity on attention to the eyes of same-race and other-race faces

Although much of the research on similarity-attraction effects has been limited to same-race contexts (typically White perceivers and White targets), several studies indicate that a similar process may occur when the target is of a different race than the perceiver. For example, early experiments that included other-race faces demonstrated that, for White participants, perceived similarity was related to more liking and a greater willingness to work with a Black or White target (Byrne & McGraw, 1964; Byrne & Wong, 1962). Likewise, research on belief congruence theory (Insko et al., 1983; Moe et al., 1981; Rokeach et al., 1960) has indicated that perceived similarities and differences in beliefs and values with a same-race or other-race target (e.g., a Black person who believes in God or a White person who is an atheist) influenced the desire to interact with the target. More recent research further revealed that perceived similarity reduced anxiety often typical in cross-race interactions and increased interest in sustained cross-race contact (West et al., 2014b).

One reason for the powerful effects of perceived similarity in an intergroup context is that people may expect to share few similarities with members of outgroups (Danyluck & Page-Gould, 2018; Mallett et al., 2008; Vorauer & Sakamoto, 2006; Vorauer et al., 1998; West et al., 2014a, 2014b). Perceiving outgroup members as different and distinct from the self is a critical component of intergroup relations (Allport, 1954). Whether we believe that members of other groups have different personality traits, physical characteristics, cultural practices, goals, or values, a lack of correspondence between me and them can have a fundamental impact on processing ingroup and outgroup members (Kawakami et al., 2017; Phills et al., 2019; Van Bavel et al., 2011). Because systemic discrimination and differential responding across racial lines is due in part to anticipated interpersonal dissimilarities between Black and White people, increasing perceived similarity between two individuals can increase attraction (Byrne & McGraw, 1964; Byrne & Wong, 1962; Hendrik & Hawkins, 1969; Robinson & Insko, 1969; Silverman & Cochrane, 1972).

It is unclear, however, whether White perceivers’ similarity with Black targets will influence attention to the eyes in the same way as explicit attraction ratings. Research by Dovidio et al. (1997) has shown that although White participants’ implicit positive attitudes and liking toward Black people predicted visual contact, explicit attitudes did not. Furthermore, although past research has demonstrated that in the very early stages of visual attention, White participants attended more to Black faces than White faces (Amodio et al., 2003; Richeson & Trawalter, 2008; Trawalter et al., 2008), in subsequent stages they preferred White faces (Van Bavel & Cunningham, 2012). In particular, initial vigilance effects in which attention was directed toward Black faces in the first instance, changed to avoidance of Black faces over longer periods (Bean et al., 2012). Similarly, experiments related specifically to attention to the eyes of same-race and other-race targets over several seconds of face processing showed that White participants attended less to the eyes of Black than White targets (Burgund, 2021; Cassidy et al., 2017; Friesen et al., 2019; Kawakami et al., 2014; Lloyd et al., 2017).

Although attention to the eyes is an important part of impression formation processes in general, it may be particularly critical in intergroup contexts. Given that cross-race interactions are often marred by misunderstandings and misperceptions (Dovidio et al., 2002; Holoien et al., 2015; Shelton & Richeson, 2006; Vorauer et al., 1998; West et al., 2014a, 2014b) and that attention to the eyes seems to play an important role in the impression formation process (Arizpe et al., 2016; Friesen et al., 2019; Itier & Batty, 2009; Kawakami et al., 2014, in press; Nguyen & Pezdek, 2017; Wang et al., 2015), investigating whether interpersonal similarity can increase eye gaze for Black targets may prove to be useful in discovering new strategies to enhance intergroup relations.

Thus, of interest in the present research was whether perceived interpersonal similarity increases attention to the eyes of both White and Black targets. Although in general, White participants have shown relatively limited attention to the eyes of Black compared to White targets, because our manipulation of similarity targeted interpersonal perceptions (i.e., how similar the target is to you) rather than intergroup perceptions (i.e., how similar are Black people to White people), it may motivate perceivers to attend closely to more similar versus less similar Black targets. Our findings, therefore, can provide insight into how perceived similarity can impact basic cognitive processes underlying positive interpersonal relations (Adams & Kleck, 2003; Baron-Cohen et al., 1997; Henderson et al., 2005; Macrae et al., 2002) across racial group boundaries.

## Overview

The primary goal of the present research was to explore the influence of perceived interpersonal similarity on visual processing of same-race and other-race faces. In two experiments, participants first completed a personality questionnaire to manipulate perceived interpersonal similarity and then viewed the faces of individuals who were ostensibly more or less similar to them, while their eye movements were tracked. The background color of the image indicated the level of similarity. The primary goal of Experiment 1 was to investigate White perceivers’ attention to the eyes of White targets as a function of perceived similarity. The aim of Experiment 2 was to replicate the results from this first study and to extend our investigation to other-race targets. Specifically, White participants were presented with faces of either White or Black targets that ostensibly varied in similarity with the participant. For both Experiments 1 and 2, we predicted a linear effect for same-race targets (i.e., White participants processing of White targets) in which greater perceived similarity would be related to more attention to the eyes. In Experiment 2, we explored whether these predicted effects also occurred for other-race faces (i.e., White participants processing of Black targets). Importantly, these experiments extend previous research on the similarity-attraction effect by investigating how interpersonal similarity influences processes related to social vision (Johnson & Adams, 2013; Johnson et al., 2015; Ofan et al., 2011; Ratner & Amodio, 2013) and add to the existing literature on top-down influences on early, subtle, attentional processes related to same-race and other-race face processing (Freeman & Ambady, 2011; Kawakami et al., 2017).

## Pilot study

To ensure that the background colors per se do not influence attention to the eyes, we conducted a pilot study. Specifically, because it is possible that people may be more likely to attend to faces or certain types of features presented on some colors more than others (Gil & Bigot, 2014; Young et al., 2013), we tested whether the background shades in the current research impacted attention to the eyes even when participants were not informed that these shades were associated with levels of similarity.

## Method

### Participants and design

To maximize power, we utilized a 4 Background Color (light to dark) × 3 Areas of Interest (AOIs: eyes, nose, mouth) within-subjects design with stimuli randomly assigned to background color. The sample sizes of previous studies investigating visual attention to ingroup and outgroup eyes have ranged from 12 to 14 per cell (Blais et al., 2008; Nakabayashi et al., 2012) to 18–20 per cell (Goldinger et al., 2009) to 21–31 per cell (Kawakami et al., 2014) to 43 per cell (Wu et al., 2012). On the basis of these latter studies, our rule for stopping data collection for all three experiments was the end of day on which we reached 40 participants. However, because the gaze patterns of some participants were difficult to track (i.e., they could not be calibrated), and the data from several participants were excluded based on a priori criteria related to familiarity with the facial stimuli and attention to the task, there are minor variations in the number of participants across experiments.

In the Pilot Study, 44 undergraduate students (28 females, *M*_age_ = 20.8 years, SD = 5.6) participated for course credit.^1^ A sensitivity analysis using MorePower 6.0.3 (Campbell & Thompson, 2012) found that our final sample could detect a two-way interaction of *η*_p_^2^ = 0.051 with 0.80 power. For both experiments, all measures, manipulations, exclusions, and crucial details related to the procedure are disclosed in the text or in the Supplementary Online Material (Additional file1). No data collection took place after analysis began.

### Procedure

After entering the laboratory, participants first completed a 44-item survey ostensibly related to personality assessment (Bernstein et al., 2007; Young & Hugenberg, 2010). Although this task was used to manipulate perceived similarity in the main studies, it had no relevance in the Pilot Study but was included so that the procedure closely matched the focal experiments. After completing the personality survey, but before starting the second task, there was a brief delay during which the computer displayed an animated icon and text stating that it was analyzing the results. Next, participants were seated in another room in front of an Eyelink monocular eye tracker (SR Research, Mississauga, Canada) with a sampling rate of 1000 Hz and presented with the eye tracking task.

Creation of Eye Tracking Stimuli. To create stimuli for the eye tracking task, headshots of students were taken at a Canadian university with a Canon PowerShot SX5 digital camera. To be consistent with recent research on attention to facial features that has used the same stimuli, we included both male and female targets (e.g., Friesen et al., 2019; Kawakami et al., 2014). We chose to include both male and female targets with neutral expressions to keep conclusions generalizable across both groups. To focus attention on internal facial features, Adobe Photoshop was used to create oval images that excluded targets’ hair. Images were also grayscaled and standardized for size (360 × 450 pixels). The mean luminance and contrast for the pictures of Black and White faces were set within a restricted range (136.20–146.96 pixels per intensity level).

**Fig. 1:**
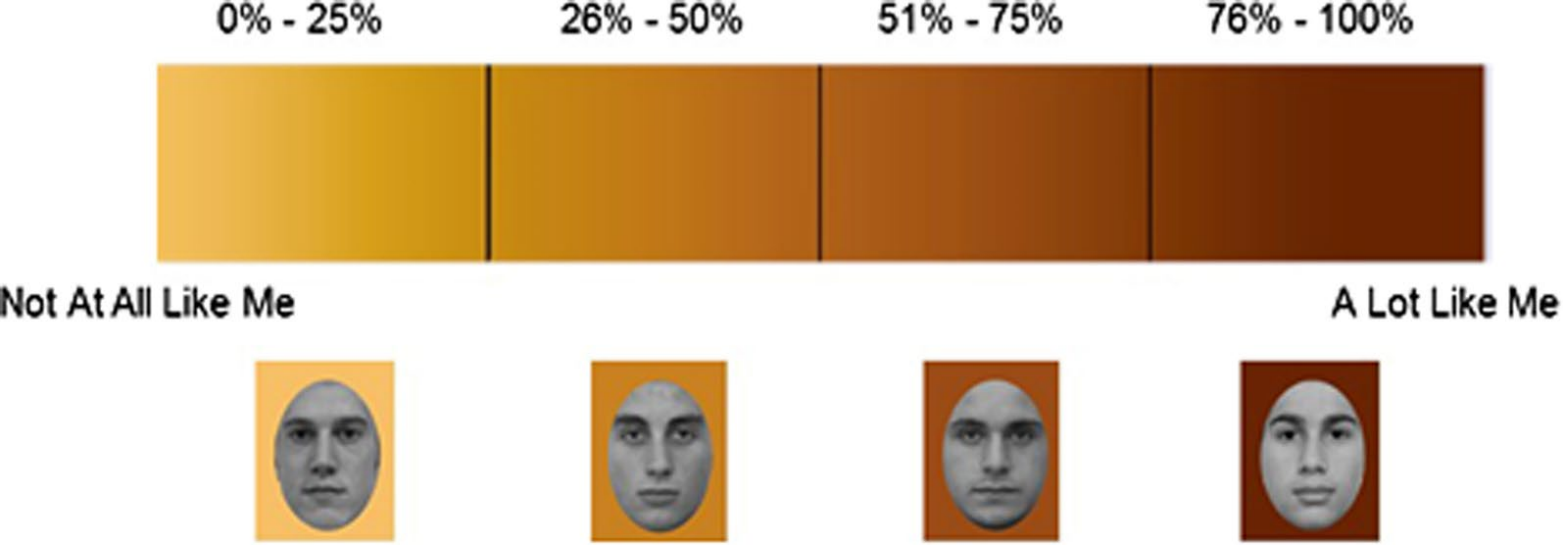
Example of the color gradient used in the instructions in Experiments 1 and 2 to manipulate perceptions of similarity between targets and participants. In the Pilot Study, the same target stimuli were presented but with no gradient or similarity information

In total, 96 White faces (half female) included in Experiments 1 and 2 and 96 Black faces (half female) included in Experiment 2 were positioned on one of four background shades from light to dark, resulting in 24 faces (12 women and 12 men) in each color level (Fig. 1). Stimuli were randomly assigned to background color when creating the set and were not randomized for individual participants. To examine whether the stimuli assigned to the background colors differed on attractiveness, perceived age, and pupil size, separate samples rated each face on a plain white background. For each characteristic, we conducted a 4 Assigned Background Color (light to dark) × 2 Race of Target (White vs. Black) mixed ANOVA with the last factor between subjects. In accordance with the paper’s main analyses, we focused on the linear effects of Assigned Background Color (see Supplementary Online Material (Additional file1) for more information on the sample, study procedures, and full results).

In the analyses related to attractiveness, only the Assigned Background Color (linear) by Race of Target interaction was significant, *F*(1, 97) = 5.51, *p* = 0.021, *η*_p_^2^ = 0.054. Simple effects analyses indicated that the linear effect for White targets was significant, *F*(1, 48) = 4.66, *p* = 0.036, *η*_p_^2^ = 0.088; targets with increasingly darker assigned background colors were rated as less attractiveness. The linear effect for Black targets, however, was not significant, *F*(1, 49) = 1.64, *p* = 0.207, *η*_p_^2^ = 0.032. In the analyses related to age, the main effect of Assigned Background Color was significant, *F*(1, 97) = 5.06, *p* = 0.027, *η*_p_^2^ = 0.050, but was qualified by the Race of Target, two-way interaction, *F*(1, 97) = 6.28, *p* = 0.014, *η*_p_^2^ = 0.061. Simple effects analyses indicated that the linear effect for White targets was significant, *F*(1, 48) = 10.48, *p* = 0.002, *η*_p_^2^ = 0.179; the estimated age of targets with increasingly darker assigned background colors was lower. The linear effect for Black targets, however, was not significant, *F*(1, 49) = 0.04, *p* = 0.852, *η*_p_^2^ = 0.001. In the analyses related to pupil size, the main effect of Race of Target was significant, *F*(1, 103) = 0.25.45, *p* < 0.001, *η*_p_^2^ = 0.198, but was qualified by a linear effect of Assigned Background Color, two-way interaction, *F*(1, 103) = 4.50, *p* = 0.036, *η*_p_^2^ = 0.042. Simple effects analyses indicated that the linear effect for White targets was significant, *F*(1, 52) = 4.21, *p* = 0.045, *η*_p_^2^ = 0.075; the pupil size of targets with increasingy darker assigned background colors was judged to be smaller. The linear effect for Black targets, however, was not significant, *F*(1, 51) = 0.82, *p* = 0.369, *η*_p_^2^ = 0.016.

In summary, while incidental differences were found between targets assigned to the varying shades of background color and the three characteristics, these differences cannot account for the predicted linear trend for similarity. Given that participants rated White targets assigned to the darkestr level as less attractive, younger, and with smaller pupil sizes and rated Black targets as not differing in attractiveness, age, or pupil size across assigned background colors, it is not likely that differences in these characteristics in targets randomly assigned to background colors determine the predicted pattern of findings. To convincingly account for our predictions that greater perceived similarity will be related to more attention to the eyes, both Black and White targets assigned to darker shades of background color would be associated with more attractiveness, younger age estimates, and larger pupil sizes.

Eye Tracking Task. In the Pilot Study, no similarity information related to the background color gradients was provided in the eye tracking task, and instructions and images were displayed on a 17-inch (43.18-cm) monitor. A chin and forehead rest minimized head movements and standardized the distance between the participant, the eye tracker (55 cm), and the display monitor (70 cm). Calibration was initially based on nine points presented twice on the screen and a single-point drift correction preceding each trial. Each trial began with a fixation cross that remained until a 1500-ms fixation registered, followed by the presentation of a single face for 5000 ms (Goldinger et al., 2009; Wu et al., 2012). To prevent habituation to stimulus location, the vertical position of the target faces varied across trials. After each face, a blank screen was presented for 1500–2000 ms. If participants did not meet the fixation cross threshold at the beginning of the trial, the target image was not displayed, and the trial was re-administered from the drift correction phase. To avoid fatigue, the trials were divided into four blocks with a break every 24 trials. In each block, participants were presented passively with three male and three female White faces on each background color. After completing the eye tracker task, participants answered demographic questions and were debriefed for suspicion.

## Results

**Fig. 2:**
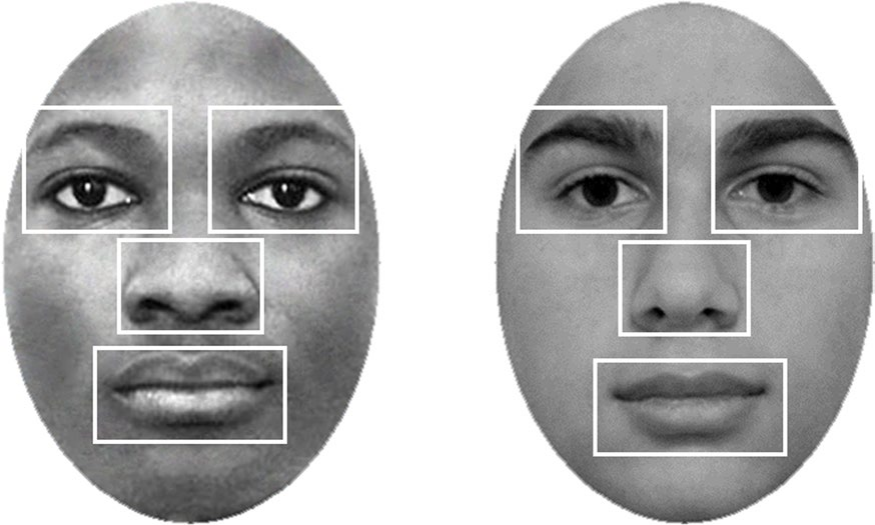
Examples of Black and White female faces with defined AOIs for the eyes, nose, and mouth

Before analyzing the data, non-overlapping AOIs for the eyes, nose, and mouth were defined (Friesen et al., 2019; Goldinger et al., 2009; Nakabayashi et al., 2012; Wu et al., 2012) that included the whole area that provided meaningful information (e.g., corners of the mouth, eyebrows), as shown in Fig. 2. The mean dwell time in milliseconds for each AOI was calculated for each background shade separately and converted into proportions of total time viewing by dividing the means by the stimulus presentation time (5000 ms).

A 4 Background Color (light to dark) × 3 AOI (eyes vs. nose vs. mouth) repeated measures ANOVA on dwell proportions produced a significant main effect of AOI, *F*(2, 86) = 125.12, *p* < 0.001, *η*_p_^2^ = 0.744, 90% CI [0.66, 0.79].^2^ In accordance with previous research (Friesen et al., 2019; Kawakami et al., 2014), simple effects analyses demonstrated that participants attended more to the eyes (*M* = 0.495, SD = 0.175) than the nose (*M* = 0.127, SD = 0.080), t(43) = 9.87, *p* < 0.001, d = 2.70, 95% CI [1.90, 3.51], and the mouth (*M* = 0.078, SD = 0.051), t(43) = 13.26, *p* < 0.001, d = 3.24, 95% CI [2.37, 4.10], and attended more to the nose than the mouth, t(43) = 4.65, *p* < 0.001, d = 0.73, 95% CI [0.40, 1.06]. The main effect of Background Color was also significant, *F*(3, 129) = 9.16, *p* < 0.001, *η*_p_^2^ = 0.176, 90% CI [0.07, 0.26], and indicated that as background colors became darker, dwell time for all features decreased, linear trend, *F*(1, 43) = 15.57, *p* < 0.001, *η*_p_^2^ = 0.266, 90% CI [0.09, 0.42].

These main effects, however, were qualified by a significant Background Color × AOI interaction, *F*(6, 258) = 5.11, *p* < 0.001, *η*_p_^2^ = 0.106, 90% CI [0.04, 0.15], see Table 1. Simple effects analyses examined the influence of Background Color on attention to each facial feature separately. Importantly, the linear trend for Background Color was not significant for attention to the eyes, *F*(1, 43) = 1.42, *p* = 0.240, *η*_p_^2^ = 0.032, 90% CI [0.00, 0.15], or attention to the nose, *F*(1, 43) = 2.58, *p* = 0.116, *η*_p_^2^ = 0.057, 90% CI [0.00, 0.19]. Unexpectedly, the linear trend for Background Color on attention to the mouth was significant, *F*(1, 43) = 17.35, *p* < 0.001, *η*_p_^2^ = 0.28, 90% CI [0.11, 0.44], such that participants attended less to mouths as the background colors became darker.

**Table 1:** Mean and standard deviations of proportion of visual attention to the eyes, nose, and mouth by background color for the Pilot Study, Experiment 1, and Experiment 2

	Background color			
	Color 1	Color 2	Color 3	Color 4
Pilot Study White faces				
Eyes Nose Mouth	.500 (.167) .138 (.090) .085 (.077)	.497 (.174) .111 (.067) .092 (.060)	.490 (.180) .140 (.100) .068 (.057)	.494 (.188) .122 (.077) .068 (.050)
Experiment 1 White faces				
Eyes Nose Mouth	.473 (.099) .112 (.050) .085 (.055)	.482 (.108) .103 (.052) .080 (.053)	.479 (.111) .110 (.052) .084 (.052)	.492 (.105) .100 (.050) .087 (.060)
Experiment 2 White faces				
Eyes Nose Mouth	.547 (.174) .155 (.093) .080 (.052)	.548 (.168) .115 (.068) .074 (.059)	.557 (.182) .122 (.088) .092 (.059)	.562 (.163) .102 (.061) .067 (.049)
Black faces				
Eyes Nose Mouth	.555 (.150) .155 (.079) .096 (.054)	.552 (.153) .128 (.096) .086 (.058)	.562 (.150) .134 (.084) .098 (.047)	.569 (.136) .121 (.081) .090 (.053)

In the Pilot Study, no information about the background color was provided. In Experiments 1 and 2, color 1 represents least similar targets with 0–25% overlap in responses on the personality survey between the participant and target, color 2 represents 26–50% overlap, color 3 represents 51%-75% overlap, and color 4 represents the most similar targets with 76–100% overlap. Standard deviations are provided in brackets

Because our primary predictions were related to greater attention to the eyes with increasing similarity, these pilot results indicated that background color per se cannot be an alternative explanation for the predicted findings. Background colors when not associated with any explicit meaning were not significantly related to greater attention to the eyes.

## Experiment 1

The aim of Experiment 1 was to investigate White perceivers’ attention to the eyes of White targets as a function of perceived similarity. In contrast to the Pilot Study, participants were informed in this experiment that the background color of each image indicated the overlap between the target and participant’s responses on the initial personality test. We predicted a linear effect for same-race targets in which greater perceived similarity would be related to more attention to the eyes.

### Method

#### Participants and design

To maximize power, we utilized a 4 Levels of Similarity (least similar to most similar) × 3 Areas of Interest (AOIs: eyes, nose, mouth) within-subjects design with stimuli randomly assigned to level of similarity. Thirty-eight White undergraduate students (25 females, M_age_ = 21.87, SD = 4.15) participated for course credit.^3^ A sensitivity analysis using MorePower 6.0.3 found that our final sample could detect a two-way interaction of *η*_p_^2^ = 0.059 with 0.80 power, which was smaller than the effect size that was obtained (*η*_p_^2^ = 0.09).

#### Procedure

Participants were first presented with the personality survey (Bernstein et al., 2007; Young & Hugenberg, 2010) used in the Pilot Study, before beginning the eye tracker task. In contrast to the Pilot Study, in the two main experiments, participants were informed that the background color of the image indicated the degree of overlap between their own responses on the survey and responses by the person depicted in the photograph. In actuality, however, target faces were randomly assigned to the background colors associated with similarity levels. Participants were presented in the eye tracker task with the 96 White faces (half female) on one of four shades described in the Pilot Study. Participants were told that over 500 other students had taken the same personality survey and that the computer had calculated the degree of personality overlap between them and these other students. Participants were informed that they would be presented with images of a subset of these students and then shown a gradient of background colors created in Adobe Photoshop (Fig. 1). They were told that the background colors represented four levels of personality similarity from “Not at all like me” to “A lot like me.” The shades represented targets that were not very similar whose test responses only overlapped up to 25% with the participant’s responses, targets whose test responses overlapped between 26% and 50%, targets whose responses overlapped between 51% and 75%, and targets that were very similar whose responses overlapped over 76% with the participant’s responses. Other than the similarity information, the procedure in the eye tracking task was the same as in the Pilot Study.

### Results and discussion

Before analyzing the data, non-overlapping AOIs for the eyes, nose, and mouth were defined and dwell proportions were calculated for each level of similarity using the same strategy as in the Pilot Study. A 4 Similarity (least to most similar) × 3 AOI (Eyes vs. Nose vs. Mouth) repeated measures ANOVA on dwell proportions produced a significant main effect of AOI, *F*(2, 74) = 279.02, *p* < 0.001, *η*_p_^2^ = 0.883, 90% CI [0.84, 0.91]. Simple effects analyses demonstrated that participants attended more to the eyes (*M* = 0.481, SD = 0.102) than the nose (*M* = 0.108, SD = 0.048), t(37) = 16.48, *p* < 0.001, d = 4.68, 95% CI [3.46, 5.90], and the mouth (*M* = 0.084, SD = 0.053), t(37) = 18.90, *p* < 0.001, d = 4.88, 95% CI [3.66, 6.11], and attended more to the nose than the mouth, t(37) = 2.31, *p* = 0.027, d = 0.47, 95% CI [0.05, 0.90]. The main effect of similarity was not significant, *F*(3, 111) = 1.48, *p* = 0.223, *η*_p_^2^ = 0.039, 90% CI [0.00, 0.09].

Importantly, the predicted two-way Similarity × AOI interaction was significant, *F*(6, 222) = 3.62, *p* = 0.002, *η*_p_^2^ = 0.089, 90% CI [0.02, 0.13], see Table 1. Simple effects analyses indicated that the linear trend for similarity on attention to the eyes was significant, *F*(1, 37) = 4.57, *p* = 0.039, *η*_p_^2^ = 0.110, 90% CI [0.003, 0.27], and that greater purported similarity was associated with more attention to the eyes. Although a significant linear trend for similarity on attention to the nose was also found, *F*(1, 37) = 8.81, *p* = 0.005, *η*_p_^2^ = 0.192, 90% CI [0.04, 0.36], greater purported similarity was associated with less, not more, attention to the nose. The linear trend for similarity on attention to the mouth was not significant, *F*(1, 37) = 0.60, *p* = 0.444, *η*_p_^2^ = 0.016, 90% CI [0.00, 0.13].

To our knowledge, these results are the first demonstration that perceived interpersonal similarity can guide visual attention to specific facial features. As expected, when targets’ responses on the personality survey were depicted as more overlapping with the participants’ responses, they attended more to the eyes of these faces. This increased attention to the eyes was not accompanied by increased attention to all facial features. Because each face was presented for 5000 ms, for participants to attend more to the eyes, they diverted attention from other facial features, such as the nose.

## Experiment 2

A primary goal of Experiment 2 was to replicate the pattern of effects in Experiment 1 for same-race faces. White participants were therefore presented with White target faces that ostensibly varied in similarity with the participant. A further aim of this study was to extend these results by investigating the impact of interpersonal similarity on the processing of other-race faces. Specifically, we examined whether purported similarity enhanced White participants’ attention to the eyes of Black targets. We predicted a linear effect for same-race and other-race faces in which greater perceived similarity would be related to more attention to the eyes.

### Method

#### Participants and design

Seventy-six White undergraduates (46 females, *M*_age_ = 20.39, SD = 4.77) completed the same procedure used in Experiment 1 with one exception, a between-subjects manipulation of target race was added.^4^ This produced a 4 Similarity (least similar to most similar) × 3 AOI (eyes, nose, mouth) × 2 Target Race (Black, White) mixed design, with the last variable between-subjects. This design provided us with the opportunity to directly replicate the results of Experiment 1 (with White targets) and to investigate whether the effects observed for same-race faces generalize to other-race Black faces. A sensitivity analysis using MorePower 6.0.3 found that our final sample could detect the predicted Similarity × AOI interaction of *η*_p_^2^ = 0.030 with 0.80 power, which was smaller than the effect size that was obtained (*η*_p_^2^ = 0.050). The minimum detectable effect size for the three-way interaction was also *η*_p_^2^ = 0.030 with 0.80 power.

#### Procedure

As in Experiment 1, all participants first completed the personality survey, followed by an eye tracker task. Half of the participants were randomly assigned to view the same set of images of White targets used in the first study and the other half viewed a new set of images of 96 Black targets. These stimuli were created, prepared, and rated using the same procedures applied to the White targets and described in the Pilot Study. Each target was randomly assigned to one of four background colors indicating personality overlap.

### Results and discussion

Before analyzing the eye tracking data, the same strategy used to define nonoverlapping AOIs for White faces was also employed for Black faces. Also, in accordance with Experiment 1, mean dwell times for the eyes, nose, and mouth of Black and White faces were calculated for each level of similarity separately and converted into proportions of total viewing time by dividing the means by the length of the presentation time (5000 ms).

A 4 Similarity (least to most similar) × 3 AOI (Eyes vs. Nose vs. Mouth) × 2 Race of Target (White vs. Black) mixed ANOVA on dwell proportions, with the last factor between subjects, produced a main effect for AOI, *F*(2, 148) = 369.20, *p* < 0.001, *η*_p_^2^ = 0.833, 90% CI [0.80, 0.87]. Replicating the results of the first study, simple effects analyses revealed that participants attended more to the eyes (*M* = 0.557, SD = 0.149) than the nose (*M* = 0.129, SD = 0.074), t(75) = 17.75, *p* < 0.001, d = 3.64, 95% CI [2.92, 4.36], and the mouth (*M* = 0.085, SD = 0.049), t(75) = 22.91, *p* < 0.001, d = 4.26, 95% CI [3.47, 5.04], and attended more to the nose than the mouth, t(75) = 5.02, *p* < 0.001, d = 0.70, 95% CI [0.41, 0.99]. The main effect of similarity was also significant, *F*(3, 222) = 18.00, *p* < 0.001, *η*_p_^2^ = 0.196, 90% CI [0.12, 0.26], and indicated that as similarity increased, dwell time for all features decreased, linear trend, *F*(1, 75) = 19.28, *p* < 0.001, *η*_p_^2^ = 0.200, 90% CI [0.08, 0.33].

These main effects, however, were qualified by the critical Similarity × AOI interaction, *F*(6, 444) = 3.86, *p* = 0.001, *η*_p_^2^ = 0.050, 90% CI [0.01, 0.07], see Table 1. As predicted, a linear trend indicated that as similarity increased, participants attended more to the eyes, *F*(1, 75) = 3.87, *p* = 0.053, *η*_p_^2^ = 0.049, 90% CI [0.00, 0.15]. A linear trend for attention to the nose was also significant, *F*(1, 75) = 55.78, *p* < 0.001, *η*_p_^2^ = 0.429, 90% CI [0.28, 0.53], such that greater perceived similarity was related to less attention to the nose. Attention to the mouth was not significantly impacted by level of similarity, *F*(1, 75) = 1.72, *p* = 0.193, *η*_p_^2^ = 0.022. Importantly, this interaction was not qualified by race. The three-way interaction was not significant, *F*(6, 444) = 0.22, *p* = 0.969, *η*_p_^2^ = 0.003, 90% CI [0.00, 0.10].

The results of Experiment 2 replicated the pattern of findings in Experiment 1, such that greater perceived interpersonal similarity was associated with more attention to the eyes. Importantly, the impact of similarity had a comparable effect on the processing of Black and White targets. Regardless of whether White participants were presented with only White or only Black faces, perceived personality overlap increased attention to the eyes.

Although past research has demonstrated that when White participants were presented with both Black and White faces, they attended more to the eyes of White than Black faces (Burgund, 2021; Cassidy et al., 2019; Friesen et al., 2019; Kawakami et al., 2014), these experiments employed a within-subjects manipulation of race, which has been shown to make race salient (Young et al., 2009). We assumed that by employing a between-subjects manipulation of race and presenting only White or only Black faces, the present design generated less of an intergroup context. In these monoracial contexts where the task focus is on interpersonal similarity, rather than between-group differences, similarity related to a purported overlap in personality affected the processing of both Black and White faces.

## General discussion

The primary goal of the present research was to investigate the impact of similarity on early perceptual processes related to same-race and other-race faces. We explored the possibility that increasing purported interpersonal similarity would lead to greater attention to the eyes. The results from two experiments supported our hypotheses. Specifically, we demonstrated that as the ostensible overlap between targets and participants on a personality questionnaire increased, White participants attended more to the eyes of White targets (Experiments 1 and 2) and Black targets (Experiment 2). Notably, target race did not moderate the impact of similarity on attention to the eyes (Experiment 2).

The present findings are in accordance with recent results related to the importance of top-down influences associated with perceiver motivation on person perception (Adams et al., 2010; Freeman & Ambady, 2011; Kawakami et al., 2017). We, however, provide a novel contribution to this literature by demonstrating how attention to facial features can be impacted by perceived similarity. When targets presumably become more similar to the perceiver, visual processing of their faces is adjusted accordingly.

Although our results replicate previous research by demonstrating that in general the eyes attract more attention than other facial features (Friesen et al., 2019; Henderson et al., 2005; Janik et al., 1978; Kawakami et al., 2014), they also show that perceived similarity can enhance this focus. Understanding the impact of perceived similarity on attention to specific facial features such as the eyes is important. Sensitivity to the eyes is found in early ERP components such as N170 amplitudes and research has shown that the eyes play a special role in person perception (Nemrodov et al., 2014). In particular, better expertise in face processing is modulated by eye gaze (Young et al., 2014) and is determined by better extraction from information in the eye region (Niedenthal et al., 2010; Vinette et al., 2004). Furthermore, other’s eyes are critical in signaling approach (Deska et al., 2016), creating feelings of connection (Wirth et al., 2010), understanding emotions (e.g., Friesen et al., 2019), and facilitating strong face representations (Burgund, 2021). Discovering ways to enhance attention to the eyes, through increasing perceived similarity, is therefore significant for improving social cognition and for better regulating interpersonal interactions. This knowledge is particularly important in an intergroup context where misperceptions, misunderstandings, and inaccurate face perception are prevalent (Kawakami et al., 2009; Page-Gould et al., 2008; Sagar & Schofield, 1980; Vorauer & Sakamoto, 2006; Vorauer et al., 1998).

The present research not only advances theorizing on face processing but also on the impact of perceived similarity. Although similarity-attraction effects are considered to be robust under certain circumstances, there are concerns about some of the paradigms employed to test this process (Condon & Crano, 1988; Montoya & Horton, 2012; Sunnafrank, 1992). One method often used to investigate this effect is the bogus stranger paradigm (Byrne, 1971; Heine et al., 2009; Rosenbaum, 1986). In this technique, participants initially complete a questionnaire related to attitudes, personality traits, or other attributes before being presented with a stranger’s responses to the same questionnaire that vary between subjects in their similarity to participants’ answers. Next, participants typically answer a single item related to liking for the target and/or a desire to work with him or her.

In contrast to this paradigm, the present method included multiple responses to multiple targets, thereby reducing concerns about stimulus sampling and scale reliability (Dierendonck, 2005; Furr, 2011; Judd et al., 2012). Furthermore, in earlier studies in which the measures of attraction were often explicit and the expected relationship between similarity and attraction was somewhat predictable, demand characteristics were a possible explanation for the effects (Sunnafrank, 1991). In the present research, alternatively, participants were not aware of the hypotheses and our measure was more indirect. Indeed, when participants were questioned about experimenter expectations after completing the study, only three students mentioned the proposed hypotheses in either experiment and these students were excluded from the analyses. These effects were therefore less vulnerable to alternative explanations related to demand characteristics and the motivated control of visual attention (Kleinke, 1986; Wellens & Faletti, 1978).

Measuring face processing in more indirect ways is especially important in an intergroup context (Greenwald & Banaji, 1995; Kawakami et al., 2017) because of strong norms against racial prejudice (Apfelbaum et al., 2008; Crandall et al., 2002; Karmali et al., 2019). Notably, in belief congruence research (Stein et al., 1965), the type of interaction (e.g., working with the target, excluding the target from my neighborhood, inviting the target home to dinner), and whether behaviors were socially sanctioned (Insko et al., 1983; Moe et al., 1981), influenced the extent to which race and perceived interpersonal similarity predicted behavioral intentions. Because of the explicit nature of the dependent variables in the latter research, people may have responded in ways that were socially acceptable and that supported the researchers’ expectations. Therefore, testing the impact of perceived similarity in paradigms in which the relationship between perceived similarity and visual attention is less obvious, and with less deliberative measures, is a valuable contribution.

It is important to note that the primary goal of Experiment 2 was to replicate the effects of the first study with White targets. A further goal was to examine whether White participants would show a similar pattern for Black targets. To fulfill both aims, we used a between-subjects design. Although the present findings may be limited to this particular monoracial context, we believe that it is important to understand if similarity affects both White and Black targets in a comparable manner. We assumed that by presenting only one race (either White or Black) to participants and focusing on interpersonal similarity (i.e., the extent to which a particular White or Black target’s responses were similar to the participant’s responses on the personality survey), race and an intergroup context would be less salient. Just as we assumed that participants would respond to White targets on an interpersonal level in Experiment 1, we proposed that a similar pattern might occur with Black targets.

However, our knowledge of determinants of interpersonal, intragroup, intergroup, and multigroup processes and how these constructs are related to stimulus presentation is limited. Impression formation processes are context dependent and identifying how people interpret their social environments is a critical next step in understanding intergroup person perception (Koch et al., 2021). For example, when people are presented sequentially in a random order with Black and White faces, do people construe their environment as more of an intergroup context than when only one race is presented? Does competition in attention to Black and White faces when presented simultaneously alter the basic pattern of attention? And how do instructions related to a focus on interpersonal relationships, such as similarity, impact the influence of these forms of presentation?

Notably, Kawakami et al. (2014) presented White participants with both Black and White targets simultaneously with no interpersonal comparisons related to similarity. Under these conditions, participants showed a clear preference for the eyes of White compared to Black faces. In a more recent study (Kawakami et al., under review), White participants were presented with the same similarity procedure in the context of a standard Own Race Bias (ORB) paradigm. In particular, in an initial learning phase, participants were presented with both Black and White targets on background colors, indicative of similarity, in a random order. Next, they completed a recognition task in which background colors were removed. Notably, in this mixed-race context, we found main effects for both race and similarity. Participants were better at recognizing their own group, White targets, than Black targets, replicating previous ORB effects. Furthermore, as level of similarity increased so did recognition of all targets. Notably, this similarity effect was not qualified by race, with a similar linear trend for both White and Black targets. Together, these findings suggest that although a context that includes the presentation of both races may increase the salience of an intergroup context and therefore may result in a greater likelihood of recognition accuracy bias across groups, it may not alter the effects of interpersonal factors, such as similarity, within groups. Although a similar pattern might be expected for attention to facial features such as the eyes, it is clear that additional research is needed using the present paradigm to test this prediction. In particular, future research should investigate the impact of similarity when participants are presented with all Black targets, all White targets, or a mix of both Black and White targets.

The personality manipulation in the current experiments has been used in the past to create an intergroup context by categorizing participants into two groups. In the present research, however, we used four levels of similarity rather than two, and the manipulation (Fig. 1) suggested a gradient of similarity between the participant and targets ranging from 0 to 100%. We assumed, with this manipulation, that rather than creating four different subgroups of similarity, participants would respond to how interpersonal similarity with a specific target impacted attention to the eyes. Although we have no data related to perceived self-similarity, the focal point of the similarity manipulation was to compare overlap between the responses of a particular target with the participant. Furthermore, a linear trend was found for similarity suggesting that incremental increases in ostensible similarity impacts attention to the eyes. Future research, however, is necessary to disentangle categorization or subgroup processes from interpersonal processes. One strategy to examine impression formation in this context would be to add additional levels of similarity. Although it is possible that participants classify targets according to four levels of subcategories, it is less likely that they would do so as the number of levels increase. This research can further inform whether there is a particular point or number of levels in which people forgo categorical processing and subgroups to respond to others on an interpersonal level. A further strategy would be to ask participants to rate overlap with each target with no background colors upon completion of the study to examine the relationship between perceptions of similarity and attention to the eyes.

One limitation of the current research is our exclusive reliance on White participants. Because of the focus on responses to male and female White and Black targets by this group, it is difficult to discern if the results are specific to these particular target and perceiver groups. In terms of the gender composition of the stimuli, in accordance with past research on face processing in an intergroup context (e.g., Friesen et al., 2019; Kawakami et al., 2014), we chose to include both male and female targets to be able to generalize our conclusions across both groups. While we did not make specific predictions about how responses might differ based on target gender in these experiments, future work should investigate whether specific gender stereotypes impact attention to the eyes of Black and White men and women.

Our decision to initially focus on Black and White racial categories was based on what we believe to be pressing issues related to the history of race relations associated with these groups in North America (Dovidio et al., 2002; Karmali et al., 2017; Kawakami et al., 2009). We advise researchers in the future, however, to recruit a variety of participants and to study a more diverse array of target social categories, to further investigate the nature of these effects. For example, would a similar pattern be found when the target groups were White and Indigenous faces? Given the recent discovery of hundreds of unmarked graves of Indigenous children who have died at residential schools in Canada (Burrage et al., 2021), examining another group that has also experienced extreme and systemic discrimination is important. Can perceived similarity increase attention to the eyes of White and Indigenous targets for White participants? Perhaps, more importantly, after years of distrust, betrayal, and animosity, can perceived similarity increase attention to the eyes of White targets for Indigenous participants? Furthermore, will this process be similar for other types of groups such as older adults, persons with disabilities, or gay men and lesbians? Will a comparable pattern also be found for minimal groups that have no history of conflictual relations?

It is important to note that this research was conducted in Canada and that each country has its own history of race relations with specific social categories and that such social and political contexts matter (Koch et al., 2021). In some environments, when racism is extreme and/or people hold more racist attitudes, people may be offended if they are informed that they are similar to a member of a stigmatized outgroup and their response may be to act in ways that distance themselves rather than create closeness. For example, Genthner et al. (1975) found that highly prejudiced White participants behaved more aggressively toward a Black confederate when they were told that their attitudes were highly similar compared to dissimilar to the confederate. Likewise, in contexts when racism is openly endorsed by authorities (Crandall et al., 2018), even manipulations of interpersonal similarity may be associated with intergroup processes, backfire, and decrease affiliative actions such as attending to the eyes of outgroups (Danyluck & Page-Gould, 2018, 2019).

The importance of perceived similarity for improving intergroup relations has a long history in social psychology. In Allport’s seminal work on The Nature of Prejudice (1954), he proposed that increased perceived similarity, driven by interpersonal contact under the right circumstances, would reduce prejudice and discrimination. The distinction between interpersonal (between two individuals) similarity and intergroup (between two groups) similarity, however, is sometimes blurred in this theorizing (Dovidio et al., 2000). Recent research indicates that intergroup similarity has a complex and inconsistent relationship with liking and positive outcomes for the outgroup (Brown & Abrams, 1986; Brown & Lopez, 2001; Danyluck & Page-Gould, 2018, 2019; Diehl, 1988; Roccas & Schwartz, 1993). Interpersonal similarity, alternatively, as proposed by the original contact theory and as suggested by our results, has the potential to reduce bias between members of distinct social categories. Although the impact of similarity on face processing in general was a primary aim of the present research, our finding that perceived interpersonal similarity between White participants and Black targets increased attention to the eyes in ways akin to White targets is notable. Whereas previous results indicate that we are attracted to all people who appear to have similar personalities and attitudes, regardless of race (Byrne & McGraw, 1964; Bryne & Wong, 1962; Insko et al., 1983; Rokeach et al., 1960), the present results suggest that these factors can also lead to comparable early person impression processes.

## Conclusion

In conclusion, although future research is clearly necessary to better understand how categorization processes and interpersonal processes interact, the present results are encouraging in that they suggest new ways through perceived similarity to enhance face processing and to improve relations between individuals from same-race and other-race categories. Because the eyes are critical to understanding others (Adams & Kleck, 2003; Baron-Cohen et al., 1997; Henderson et al., 2005; Macrae et al., 2002), this work offers important insights into potential interventions related to perceived similarity to decrease miscommunication and misperceptions between races and to facilitate social interactions (Guéguen et al., 2011; Walton et al., 2012; West et al., 2014a, 2014b).

## Open practices statement

The datasets supporting the conclusions for the Pilot Study, Experiment 1, and Experiment 2 are available at the Open Science Framework (OSF): https://osf.io/k9jx3/ None of the experiments were preregistered.

## Supplementary Information

**Additional file 1**. Supplemental Online Material.

## Data Availability

The datasets supporting the conclusions for the Pilot Study, Experiment 1, and Experiment 2 are available at the Open Science Framework (OSF): https://osf.io/k9jx3/

## Footnotes

1 The data from two additional participants were collected but not included in the analyses because they reported personally recognizing some of the experimental targets, which were photographs of students at their university.

2 Effect size confidence intervals for η_p_^2^ were calculated using the SPSS macros provided by Wuensch (2016a, 2016b), Per Steiger (2004), and Lakens (2014); we report 1 – α CIs for Cohen’s *d* and 1—2α CIs around η_p_^2^. CIs around Cohen’s *d* are approximate as described by Kline (2013, pp. 142–143).

3 The data from seven additional participants were collected but not included in the analyses because they reported personally recognizing some of the experimental targets (*n* = 5) or based on research assistants’ notes related to a lack of attention to the task (*n* = 2).

4 Although the data from eight additional participants were collected, they were not included in the analyses because the participants did not attend to the task or instructions as noted by research assistants (*n* = 2), reported awareness of the study’s hypotheses (*n* = 3), or reported they personally recognized some of the experimental targets (*n* = 3).
