# Supplementary material for: Impact of perceived interpersonal similarity on attention to the eyes of same-race and other-race faces
Source: Cogn Res Princ Implic. 2021 Nov 2;6:68. doi: 10.1186/s41235-021-00336-8 (PMC8563912; doi:10.1186/s41235-021-00336-8)
Supplement: Supplementary file 1 — Additional file 1. Supplemental Online Material. [file 41235_2021_336_MOESM1_ESM.docx]

**Supplementary Online Materials**

**Creation of Eye Tracking Stimuli**

**Method & Procedure**

To create stimuli for the eye tracking task, headshots of students were taken at a Canadian university with a Canon PowerShot SX5 digital camera. To be consistent with recent research on attention to facial features that has used the same stimuli, we included both male and female targets (e.g., Friesen et al., 2019; Kawakami et al., 2014). To focus attention on internal facial features, Adobe Photoshop was used to create oval images that excluded the target’s hair. Images were also grayscaled and standardized for size (360 X 450 pixels). The mean luminance and contrast for the pictures of Black and White faces was set within a restricted range (136.20–146.96 pixels per intensity level). In total, 96 White faces (half female) on one of four background shades from light to dark, resulting in 24 faces (12 women and 12 men) in each color level. Stimuli were randomly assigned to background color when creating the set and were not randomized for individual participants.

To examine whether the stimuli assigned to the background colors differed on attractiveness, perceived age, and pupil size, separate samples rated the faces on a white background. To maintain consistency with participants’ experience within the experiments, participants rated either Black or White photos, between-subjects. To reduce task length, in each sample of participants rated a random selection of half the total stimuli, and stimuli order was randomized by individual. Each stimulus was therefore rated by ~25 individuals.

Participants were nonBlack Canadian adults recruited using Prolific Academic (http://prolific.co). One group (*N* = 99) used 7-point scales to rate stimuli attractiveness (ranging from *not at all* to *extremely*) and age in 5-year increments (1=16-20, 2=21-25… 7=older than 45). Another group (*N* = 105) rated stimuli pupil size on a 5-point scale with the labels 1=*Very constricted (small)*, 2=*Somewhat constricted*, 3=*Neither or can’t tell*, 4=*Somewhat dilated*, 5=*Very dilated (big)*.

**Results**

For each characteristic, we conducted a 4 Assigned Background Color (light to dark) × 2 Race of Target (White vs. Black) mixed ANOVA with the last factor between-subjects. In accordance with the paper’s main analyses, we focused on the linear effects of similarity. In sum, although the photos were randomly assigned to similarity level, some differences in these ratings still emerged.

In the analyses related to attractiveness, only the Assigned Background Color (linear) by Race of Target interaction was significant, F(1, 97) = 5.51, p = .021, η_p_^2^ = .054. Simple effects analyses indicated that the linear effect for White targets was significant, F(1, 48) = 4.66, p = .036, η_p_^2^ = .088, with increasingly darker assigned background colors, targets were rated as less attractiveness. The linear effect for Black targets, however, was not significant, F(1, 49) = 1.64, p = .207, η_p_^2^ = .032. In the analyses related to age, the main effect of Assigned Background Color was significant, F(1, 97) = 5.06, p = .027, η_p_^2^ = .050, but was qualified by the Race of Target, two-way interaction, F(1, 97) = 6.28, p = .014, η_p_^2^ = .061. Simple effects analyses indicated that the linear effect for White targets was significant, F(1, 48) = 10.48, p = .002, η_p_^2^ = .179, the estimated age of targets with increasingly darker assigned background colors was lower. The linear effect for Black targets, however, was not significant, F(1, 49) = .04, p = .852, η_p_^2^ = .001. In the analyses related to pupil size, the main effect of Race of Target was significant, F(1, 103) = .25.45, p < .001, η_p_^2^ = .198, but was qualified by a linear effect of Assigned Background Color, two-way interaction, F(1, 103) = 4.50, p = .036, η_p_^2^ = .042. Simple effects analyses indicated that the linear effect for White targets was significant, F(1, 52) = 4.21, p = .045, η_p_^2^ = .075, the pupil size of targets with increasingly darker was judged to be smaller. The linear effects for Black targets, however, was not significant, F(1, 51) = .82, p = .369, η_p_^2^ = .016.

Table 1: Rated means (SD) of stimuli attractiveness

|  | Assigned Background Color | | | |
| --- | --- | --- | --- | --- |
|  | 1 | 2 | 3 | 4 |
| Black Targets | 3.35 (.94) | 3.42 (.92) | 3.34 (.90) | 3.48 (.98) |
| White Targets | 3.43 (.78) | 3.41 (.76) | 3.48 (.82) | 3.27 (.80) |

Table 2: Rated means (SD) of stimuli estimated age

|  | Assigned Background Color | | | |
| --- | --- | --- | --- | --- |
|  | 1 | 2 | 3 | 4 |
| Black Targets | 2.99 (.70) | 3.01 (.79) | 3.12 (.67) | 2.96 (.68) |
| White Targets | 3.14 (.55) | 2.84 (.59) | 3.05 (.54) | 2.87 (.57) |

Table 3: Rated means (SD) of stimuli pupil size

|  | Assigned Background Color | | | |
| --- | --- | --- | --- | --- |
|  | 1 | 2 | 3 | 4 |
| Black Targets | 2.99 (.43) | 3.05 (.53) | 2.83 (.47) | 3.12 (.46) |
| White Targets | 3.37 (.40) | 3.52 (.44) | 3.17 (.41) | 3.35 (.53) |

**Discussion**

In summary, while incidental differences were found between varying shades of background color and the three characteristics, these differences cannot account for the predicted linear trend for similarity. Given that participants rated White targets assigned to the darkest level as less attractive, younger, and with smaller pupil sizes and rated Black targets as not differing in attractiveness, age, or pupil size across assigned background colors, it is not likely that differences in these characteristics in targets randomly assigned to background colors determine the predicted pattern of findings. To convincingly account for our predictions that greater perceived similarity will be related to more attention to the eyes, both Black and White targets assigned to darker shades of background color would be associated with more attractiveness, younger age estimates, and larger pupil sizes.

**Supplementary Analysis in Experiment 2**

After completing the eye-tracking task in Experiment 2, participants were also instructed to complete an exploratory partner choice task. Specifically, on each trial, participants were presented with four faces from the eye tracking task in a quadrant and were asked to choose a partner for a future hypothetical task. In these quadrants, one face was presented from each similarity level, however, the background colors were removed. In total, participants completed 24 trials. A mixed ANOVA on partner choice frequencies produced a significant Similarity X Race of Target interaction, *F*(3, 222) = 4.67, *p* = .003, η^2^_p_ = .06, 95% CI[.01, .11]. Simple effects analyses for White targets demonstrated a linear trend in which as similarity increased, participants chose *fewer* (not more) White targets as partners, *F*(1, 40) = 7.26, *p* = .01, η^2^_p_ = .15, 95% CI[.02, .32]. Alternatively, for Black targets, the linear trend was not significant, *F*(1, 34) = .76, *p* = .39, η^2^_p_ = .02, 95% CI[.00, .15]. Given that participants may have had difficulty in remembering the level of similarity associated with each of the 96 faces once the background color was removed, we are not confident that these results are meaningful or can be replicated.

**References**

Friesen, J. P., Kawakami, K., Vingilis-Jaremko, L., Caprara, R., Sidhu, D. M., et al. (2019). Perceiving happiness in an intergroup context: The role of race and attention to the eyes in differentiating between true and false smiles. *Journal of Personality and Social Psychology*, *116*, 375–395. doi:10.1037/pspa0000139

Kawakami, K., Williams, A., Sidhu, D., Choma, B. L., Rodriguez-Bailón, R., et al. (2014). An eye for the I: Preferential attention to the eyes of ingroup members. *Journal of Personality and Social Psychology*, *107*, 1–20. doi:10.1037/a0036838
